# Retrospective, multicenter study of surgical treatment for carotid body tumors with or without preoperative embolization

**DOI:** 10.3389/fonc.2023.1123430

**Published:** 2023-03-02

**Authors:** Tonglei Han, Jiaxi Pu, Hanfei Tang, Shaofei Yang, Dandan Dong, Minhao Lu, Xiaolong Wei, Guanghua Yang, Bin Zhao, Daqiao Guo, Xiao Tang, Zhiqing Zhao

**Affiliations:** ^1^ Department of Vascular Surgery, Zhongshan Hospital, Fudan University, Shanghai, China; ^2^ Department of General Surgery, Seventh People's Hospital of Shanghai University of Traditional Chinese Medicine, Shanghai, China; ^3^ Department of Vascular Surgery, Changhai Hospital, Naval Military Medical University, Shanghai, China

**Keywords:** carotid body tumor, surgical resection, preoperative embolization, stroke, cranial nerve injuries

## Abstract

**Background:**

Carotid body tumor (CBT) is the most common head and neck paraganglioma. Whether preoperative embolization benefits CBT patients who will receive surgical resection is still controversial.

**Methods:**

In this multi-center retrospective study, we collected data from patients with CBT who received surgical treatment without (group A) or with preoperative embolization (group B) from 2011 to 2019. The primary outcome was the rate of death or stroke after 3 years of follow-up. The secondary outcomes of the study were length of operation (LOO), intraoperative blood loss (IBL), length of stay (LOS), rate of recurrence, and rate of cranial nerve (CN) injuries. Descriptive statistics were used to analyze the demographics, clinical characteristics, complications, and follow-up results of the patients.

**Results:**

Between January 2011 and October 2019, 261 consecutive patients (107 male and 154 female) entered analysis. After 3 years of follow-up, no patient died in both groups. Only three patients with stroke were detected: 2/226 (0.9%) in group A vs. 1/35 (2.9%) in group B (p = .308). The LOO in group A was 132.6 ± 64.6 min compared with 152.9 ± 40.4 min in group B (p = .072). IBL in group A was 375.4 ± 497.8 ml compared with 448.0 ± 270.8 ml in group B (p = .400). LOS in group A was 8.3 ± 2.0 days compared with 7.4 ± 1.7 days in group B (p = .016). Seventy-two CN injuries were detected: 65/226 (28.8%) in group A vs. 7/35 (20.0%) in group B (p = .281). There were 65 temporary CN injuries (59 in group A vs. 6 in group B) (p = .254) and seven permanent CN injuries (6 in group A vs. 1 in group B) (p = .945). Three most frequently injured cranial nerves were the pharyngeal branch and superior laryngeal nerve (12.3%), recurrent laryngeal nerve (7.7%) and vagus nerve (7.3%).

**Conclusion:**

There was insufficient evidence to support the efficacy of preoperative embolization. CBT resection alone had a similar rate of stoke, recurrence, and CN injuries when compared with CBT resection with preoperative arterial embolization. Meanwhile, CBT resection alone did not increase LOO and IBL.

## Introduction

Carotid body tumor (CBT) is a rare tumor of the neck that constitutes 60% of head and neck paraganglioma with a relative low incidence (1:30,000) (1–4). This carotid bifurcation originated tumor frequently causes neck masses and sometimes makes damage to the nerves and vessels within or close to it, including the vagal nerve, recurrent laryngeal nerve, hypoglossal nerve, glossopharyngeal nerve, or carotid vessels (5, 6). Patients generally present with a lateral slowly enlarging painless neck mass and only a part have accompanying odynophagia or dysphagia related to compression. Although the incidence of malignant transformation in CBTs is less than 10%, surgery remains the best choice of treatment for patients with appropriate operative risk.

Multiple challenges exist in CBT surgical resection because of the robust vascularity and complicated anatomic location. In most cases, the CBT receives its blood supply from the external carotid artery. Hence, several studies suggested that preoperative embolization might be an alternative method for preventing blood loss before surgery treatment (4, 7–10). However, the preoperative embolization lengthens the overall treatment time of CBT, increasing the economic burden on patients as well. Recent studies indicated that preoperative embolization did not reduce operative time or intraoperative blood loss significantly (11, 12), and it might even increase the risk of stroke in CBT patients (13). There is still some controversy in regard to whether this approach is beneficial or not (11, 14, 15). Most of the research studies on this topic consist of small single-center retrospective reviews without a definitive conclusion. The objective of this multicenter study is to determine the effect of preoperative embolization on blood loss, postoperative neurologic events, and inpatient mortality.

## Methods

### Inclusion and exclusion criteria

Patients who underwent CBT surgical resection with or without preoperative embolization in Shanghai Seventh People’s Hospital (Shanghai, China), Zhongshan Hospital (Shanghai, China), and Changhai Hospital (Shanghai, China) from January 2011 to October 2019 were enrolled in this retrospective study. The following patients were excluded: 1) only a part of larger CBTs, like Shamblin II and III, were suitable for preoperative embolization, so patients with Shamblin I CBT were excluded; 2) to avoid the influence of other confounding factors and explore whether preoperative embolization is a direct risk factor associated with post-operative stroke, patients who had carotid artery reconstructions, including resections, stenting, artificial vascular replacement or bypass surgery intra-operative were excluded; 3) a previous history of ipsilateral neck irradiation or surgery might damage the local vessels and nerves and cause a certain bias, so these patients were also excluded; 4) to verify the outcomes of preoperative embolization, CBT patients with pre-operative clinical symptoms were excluded.

### Population data

Each enrolled patient underwent routine preoperative computed tomography (CT) and was grouped according to previous treatment: CBT resection alone (group A) and CBT resection with preoperative arterial embolization (group B).

This trial was approved by the Ethics Committee and Institutional Review Board of Shanghai Seventh People’s Hospital, Zhongshan Hospital, and Changhai Hospital. All patients’ with written informed consent form were collected. The pre-embolization and surgical resection procedures were performed by three trained experienced vascular surgeons in three included centers.

### Pre-embolization procedure

Under local anesthesia, a preoperative embolization procedure was performed 1–2 days prior to the surgical resection of CBTs. We introduced a 6-French (Fr) sheath into the femoral artery. And then a 5-Fr guide catheter was advanced into the common carotid artery. The following digital subtraction angiography (DSA) was performed to know the details of the internal and external carotid artery: Under roadmap, a microcatheter was introduced into the tumor-feeding artery through the guide catheter. Subsequently, embolization was carried out using gelatin microsphere particles ranging from 100 to 500 mm. To assess the effectiveness of embolization and ICA patency, a final angiogram was performed. The patients were given 100 IU/kg of low-molecular-weight heparin during the procedure. No anticoagulant or antiplatelet agents were used due to the following surgical resection procedure.

### Surgical resection procedure

Surgery was performed under general anesthesia with identification of the internal jugular vein and exposure of the superior thyroid artery in each patient. Ligation was performed on these common veins that would impede surgery. The decision to repair the vessel intraoperatively was made by two experienced vascular surgeons on the basis of clinical experience and strict technical standards. For the prevention of intraoperative cerebral ischemia, the common carotid artery was not blocked in most CBT surgeries. Generally, the superior laryngeal nerve accompanying the superior thyroid artery and the vagus nerve accompanying the common carotid artery can be observed. The hypoglossal nerve was also visible during some operations. All nerves should be preserved in principle. The CBTs were finally dissected and successfully removed.

### Definition

CBTs were classified into different Shamblin types according to a previous report (16). Shamblin I CBTs are relatively small, barely attaching to the carotid vessels. Surgical excision of these tumors is easily performed. Shamblin II CBTs refer to larger tumors that are moderately encroaching on the carotid vessels and can be surgically removed carefully. Shamblin III CBTs are characterized by large tumors that completely surround the carotid arteries. Removing this type of CBT is more risky and challenging. Preoperative CTA is used as the basis for determining CBTs’ Shamblin type. All related medical records of CBT patients were reviewed, including preoperative patients’ profiles, intraoperative findings, and 3 years of follow-up conditions. The contrast-enhanced CTA and Duplex ultrasound performed at 3 and 12 months and 2 and/or 3 years were evaluated. The telephone interviews were conducted at the sixth month, first, second, and third year.

The primary outcome was the rate of death or stroke after 3 years follow-up. The secondary outcomes of the study were length of operation (LOO), intraoperative blood loss (IBL), length of stay (LOS), the rate of recurrence, and the rate of cranial nerve (CN) injuries. The assessment of CN function was conducted based on clinical symptoms. These symptoms that still existed 2 years postoperative were regarded as permanent CN injuries and not included in the temporary ones. Temporary CN injury symptoms after CBT surgery included (1) transient ischemic attack (dizziness, headache, and temporary blurred vision), (2) tongue bias, (3) dysphagia, (4) hoarseness, and (5) eyelid ptosis.

### Statistical analysis

Continuous variables were reported as mean ± standard deviation (SD). Skewed variables were summarized as median and range, depending on the distribution of the variables. Group comparisons were analyzed with Student’s *t*-test or Wilcoxon rank-sum test for numerical variables and χ^2^ or Fisher’s exact test for categorical variables.

Uni- and multivariable analyses on the main outcomes at different follow ups were performed through logistic regressions to attest the group A vs. group B OR and evaluate effects of other variables. A decision tree analysis was performed to determine the factors that predict which patients may benefit from preoperative embolization. All analyses were performed using Empower (R) (www.empowerstats.com, X&Y solutions, Inc., Boston, MA, USA) and R (http://www.R-project.org). A p-value less than 0.05 was considered statistically significant.

## Results

### Study population

**Figure 1:**
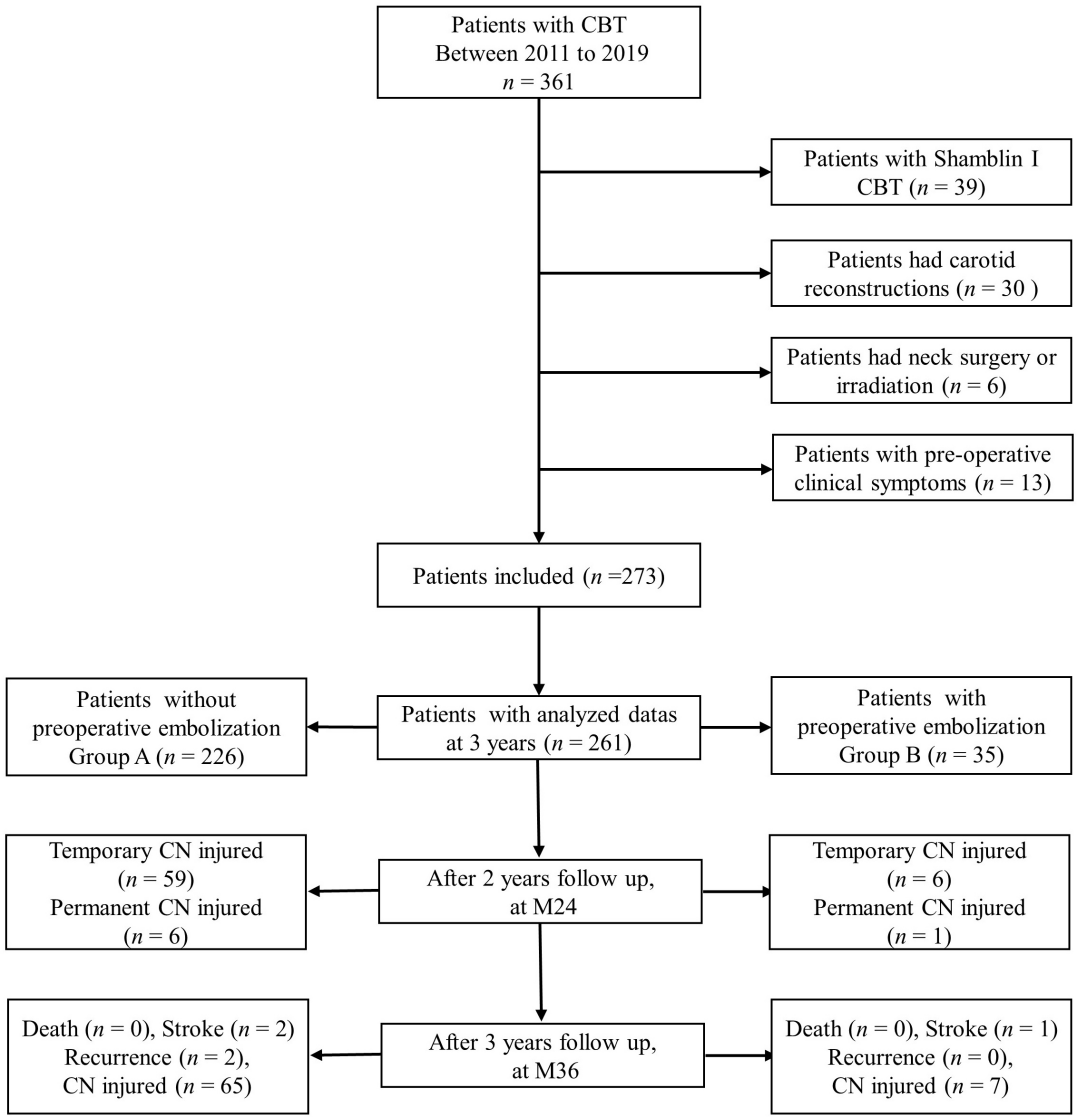
The flow diagram of this multicenter retrospective study for CBT patients with or without preoperative embolization after 3 years of follow-up. CBT, Carotid body tumor; CN, cranial nerve.

In the current study, 261 consecutive patients (107 male and 154 female) entered analysis. The flow chart is detailed in **Figure 1**. Mean patients’ age was 43.7 ± 13.0 years (range, 10–77 years). Besides, 148 tumors were located on the left side and 112 tumors located on the right. Tumors were classified using the Shamblin system as follows: Shamblin type II (112, 43.4%) and Shamblin type III (146, 56.6%). The characteristics of patients and tumors are shown in **Table 1**.

**Table 1:** Baseline demographics and characteristics for patients with CBT surgical resection alone (group A) or with preoperative embolization (group B).

Patient demographics	Total (n=261)	Group A (n=226)	Group B (n=35)	P-value	Test
Male, gender	107 (41.0%)	94 (41.6%)	13 (37.1%)	0.618	a
Age, years	43.7 ± 13.0	43.8 ± 12.5	42.8 ± 16.1	0.675	b
Body mass index, kg/m^2^	22.8 ± 3.3	22.5 ± 3.1	24.4 ± 4.0	0.001	b
Shamblin type				<0.001	a
II	112 (43.4%)	109 (48.9%)	3 (8.6%)		
III	146 (56.6%)	114 (51.1%)	32 (91.4%)		
Thyroid disease	10 (3.8%)	7 (3.1%)	3 (8.6%)	0.116	a
Family history	1 (0.4%)	1 (0.4%)	0 (0.0%)	0.693	a
Hypertension	35 (13.4%)	29 (12.8%)	6 (17.1%)	0.486	a
Diabetes	11 (4.2%)	11 (4.9%)	0 (0.0%)	0.182	a
Coronary heart disease	5 (1.9%)	5 (2.2%)	0 (0.0%)	0.374	a
Drinking				0.183	a
Never	199 (76.2%)	168 (74.3%)	31 (88.6%)		
Current	17 (6.5%)	16 (7.1%)	1 (2.9%)		
Previous	45 (17.2%)	42 (18.6%)	3 (8.6%)		
Smoking				0.366	a
Never	218 (83.5%)	186 (82.3%)	32 (91.4%)		
Current	21 (8.0%)	20 (8.8%)	1 (2.9%)		
Previous	22 (8.4%)	20 (8.8%)	2 (5.7%)		

CBT, carotid body tumor.

Data are presented as n (%) or mean ± standard deviation unless stated otherwise.

*p values at a = .05 were considered statistically significant.

a, chi-square test; b, Student t test;

### Technical success

The procedural success rate of selective embolism was 100% in group B. Complete resection of the CBT was achieved in 261 cases (100%).

### Outcomes

#### Primary outcome

After 3 years of follow-up, no patient died in both groups. Only three patients with stroke were detected: 2/226 (0.9%) in group A vs. 1/35 (2.9%) in group B (p = .308). These independent risk factors were selected by using uni- (**Table 2**) and multivariable analysis (**Table 3**), including age, gender, BMI, Shamblin type, thyroid disease, drinking. The estimated OR for group B with group A as the baseline group was 3.3 (95% CI 0.3–37.3) (p = .336) and the adjusted OR (with age, female, BMI, Shamblin type, thyroid disease, and drinking as covariables) was 2.0 (95% CI 0.1–38.5) (p = .655). In these three patients with stroke, one in group A had clopidogrel resistance and ticagrelor was used as a substitute drug. Despite this, he developed acute stoke within 30 days postoperative due to the embolism of the common carotid artery. Unfortunately, no clear etiology was found in the other two stroke patients.

**Table 2:** Univariate regression analysis for primary and secondary outcomes.

Univariate	Stroke	Recurrence	CN injuries	LOO	IBL	LOS
Age, years	0.9 (0.8, 1.0) 0.054	1.0 (0.9, 1.1) 0.884	1.0 (1.0, 1.0) 0.458	**-0.7 (-1.3, -0.1) 0.023**	**-6.6 (-11.0, -2.2) 0.004** 0.0040.024	-0.0 (-0.0, 0.0) 0.256
Gender						
Male	1.0	1.0	1.0	0	0	0
Female	1.4 (0.1, 15.6) 0.787	——	0.8 (0.4, 1.3) 0.327	**-21.3 (-36.6, -6.0) 0.007**	**-135.1 (-251.5, -18.7) 0.024**	**-0.6 (-1.1, -0.1) 0.025**
Body mass index, kg/m2	0.8 (0.6, 1.2) 0.395	0.9 (0.6, 1.4) 0.697	0.9 (0.8, 1.0) 0.076	**2.4 (0.0, 4.7) 0.048**	2.5 (-15.6, 20.6) 0.786	**-0.1 (-0.2, -0.0) 0.019**
Shamblin type						
II	1.0	1.0	1.0	0	0	0
III	——	0.8 (0.0, 12.4) 0.851	1.1 (0.6, 1.9) 0.695	12.3 (-3.0, 27.6) 0.116	**147.8 (31.6, 264.0) 0.013**	0.0 (-0.4, 0.5) 0.895
Thyroid disease	**13.8 (1.1, 167.0) 0.039**	——	0.6 (0.1, 3.1) 0.587	-6.5 (-45.9, 32.9) 0.747	-49.1 (-349.1, 250.9) 0.748	-0.7 (-2.0, 0.6) 0.289
Drinking						
Never	1.0	1.0	1.0	0	0	0
Current	——	——	**3.2 (1.2, 8.7) 0.024**	15.9 (-14.9, 46.8) 0.313	69.2 (-166.3, 304.7) 0.565	-0.5 (-1.5, 0.5) 0.322

CN, cranial nerve; LOO, length of operation; IBL, intraoperative blood loss; LOS, length of stay.

Data are presented as OR (95% CI), P or beta (95% CI), P.

*p values at a = .05 were considered statistically significant;

——indicates meaningless.

Bold represents statistical significance.

**Table 3:** Multivariable regression analysis for primary and secondary outcomes.

Multivariable	Stroke	Recurrence	CN injuries	LOO	IBL	LOS
Age, years	0.8 (0.6, 1.1) 0.124	1.0 (0.8, 1.3) 0.730	1.0 (1.0, 1.0) 0.633	**-1.0 (-1.6, -0.3) 0.003**	**-6.8 (-11.6, -2.1) 0.005**	-0.0 (-0.0, 0.0) 0.514
Gender						
Male	1.0	1.0	1.0	0	0	0
Female	1.4 (0.0, 80.3) 0.866	——	0.8 (0.4, 1.5) 0.486	-16.9 (-34.2, 0.5) 0.057	-129.6 (-260.8, 1.6) 0.054	**-0.5 (-1.1, -0.0) 0.048**
Body mass index, kg/m2	1.0 (0.6, 1.5) 0.860	0.2 (0.0, 7.2) 0.386	0.9 (0.8, 1.0) 0.054	**3.4 (1.0, 5.8) 0.007**	12.2 (-6.4, 30.8) 0.199	**-0.1 (-0.2, -0.0) 0.013**
Shamblin type						
II	1.0	1.0	1.0	0	0	0
III	——	0.5 (0.0, 461.3) 0.827	1.2 (0.7, 2.2) 0.521	**15.7 (0.3, 31.0) 0.046**	**155.7 (39.1, 272.4) 0.009**	-0.1 (-0.6, 0.3) 0.596
Thyroid disease	**373.7 (1.9, 74963.8) 0.029**	——	1.0 (0.2, 5.0) 0.972	6.0 (-33.1, 45.2) 0.763	65.2 (-231.4, 361.8) 0.667	-0.2 (-1.5, 1.0) 0.694
Drinking						
Never	1.0	1.0	1.0	0	0	0
Current	——	——	0.8 (0.3, 2.6) 0.766	17.4 (-12.3, 47.2) 0.252	**240.6 (14.9, 466.2) 0.038**	0.8 (-0.1, 1.8) 0.075

CN, cranial nerve; LOO, length of operation; IBL, intraoperative blood loss; LOS, length of stay.

Data are presented as OR (95% CI), P or beta (95% CI), P.

*p values at a = .05 were considered statistically significant;

Adjust model adjusted for: age, gender, BMI, Shamblin type, thyroid disease, drinking.

——indicates meaningless.

Bold represents statistical significance.

#### Secondary outcomes

##### LOO, IBL, and LOS

The baseline procedural characteristics are described in **Table 4**. The total LOO in all patients was 135.3 ± 62.2 min: 132.6 ± 64.6 min in group A and 152.9 ± 40.4 min in group B; the estimated beta for group B with group A as the baseline group was 20.3 (95% CI -1.7 to 42.4) (p = .072) and the adjusted beta was 10.2 (95% CI 0.3–1.8) (p = .435).

**Table 4:** Technical data of CBT surgical resection procedure for patients without (group A) or with preoperative embolization (group B).

CBT surgical resection procedure	Total (n=261)	Group A (n=226)	Group B (n=35)	P-value	Test
Tumor location				0.459	a
Left	149 (57.1%)	127 (56.2%)	22 (62.9%)		
Right	112 (42.9%)	99 (43.8%)	13 (37.1%)		
LOO, min	135.3 ± 62.2	132.6 ± 64.6	152.9 ± 40.4	0.072	b
IBL, ml	385.2 ± 473.7	375.4 ± 497.8	448.0 ± 270.8	0.400	b
LOS, day	8.2 ± 2.0	8.3 ± 2.0	7.4 ± 1.7	**0.016**	b

CBT, carotid body tumor; LOO, length of operation; IBL, intraoperative blood loss; LOS, length of stay.

Data are presented as n (%) or mean ± standard deviation unless stated otherwise.

*p values at a = .05 were considered statistically significant.

a, chi-square test; b, Student t test;

Bold represents statistical significance.

The IBL in both groups was 385.2 ± 473.7 ml: 375.4 ± 497.8 ml in group A and 448.0 ± 270.8 ml in group B; the estimated beta for group B with group A as the baseline group was 72.6 (95% CI -96.3 to 241.5) (p = .400) and the adjusted beta was -22.5 (95% CI -201.8 to 156.8) (p = .806).

The LOS in all patients was 8.2 ± 2.0 days: 8.3 ± 2.0 day in group A and 7.4 ± 1.7 days in group B; the estimated beta for group B with group A as the baseline group was -0.9 (95% CI -1.6 to -0.2) (p = .016) and the adjusted beta was -0.7 (95% CI -1.4 to 0.0) (p = .069).

##### The rate of recurrence and CN injuries

After 3 years of follow-up, there were only two recurrences in group A and none in group B, no significant differences were observed between 2 groups (p = .576).

The statistics of temporary and permanent CN injuries in both groups are shown in **Table 5**. Seventy-two CN injuries were detected: 65/226 (28.8%) in group A vs. 7/35 (20.0%) in group B (p = .281). The estimated OR for group B with group A as the baseline group was 0.6 (95% CI 0.3–1.5) (p = .284), and the adjusted OR was 0.7 (95% CI 0.3–1.8) (p = .435).

**Table 5:** Temporary and permanent CN injuries in both groups.

CN injuries	Temporary		P-value	Permanent		P-value
	Group A (n=226)	Group B (n=35)		Group A (n=226)	Group B (n=35)	
Pharyngeal branch and superior laryngeal nerve	28 (12.4%)	4 (11.4%)	0.872	2 (0.9%)	1 (2.9%)	0.308
Vagus nerve	15 (6.6%)	4 (11.4%)	0.310	0 ( 0.0%)	0 ( 0.0%)	——
Accessory nerve	6 (2.7%)	1 (2.9%)	0.945	0 ( 0.0%)	0 ( 0.0%)	——
Hypoglossal nerve	9 (4.0%)	4 (11.4%)	0.060	6 (2.7%)	1 (2.9%)	0.945
Recurrent laryngeal nerve	16 (7.1%)	4 (11.4%)	0.368	1 (0.4%)	0 ( 0.0%)	0.693
Sympathetic ganglion	13 (5.8%)	0 ( 0.0%)	0.145	0 ( 0.0%)	0 ( 0.0%)	——

CN, cranial nerve.

Data are presented as n (%).

*p values at a = .05 were considered statistically significant;

Student t test.

——indicates meaningless.

In these CN injuries, there were 65 temporary CN injuries (59 in group A vs. 6 in group B) (p = .254); the estimated OR for group B with group A as the baseline group was 0.6 (95% CI 0.2–1.5) (p = .258) and the adjusted OR was -0.0 (95% CI -0.2 to 0.1) (p = .562). Seven permanent CN injuries (six in group A vs. one in group B) (p = .945); the estimated OR for group B with group A as the baseline group was 1.1 (95% CI 0.1–9.2) (p = .945) and the adjusted OR was -0.0 (95% CI -0.1 to 0.0) (p = .633).

Pharyngeal branch and superior laryngeal nerve (12.3%), recurrent laryngeal nerve (7.7%), and vagus nerve (7.3%) were the most frequently injured CNs after CBT resection. Above all, 57 patients had at least one CN injury in the current study, and 24 patients had multiple CN injuries.

##### Decision tree analysis

The decision tree analysis was performed as previously reported (11). The five most important variables for distinguishing patients who should receive preoperative embolization were BMI, Shamblin III, age, current drinking, and female sex. The variable importance is shown in **Table 6**. Larger values indicate a greater difference between the two subgroups in terms of the prevalence of the dependent variable.

**Table 6:** Based on decision tree analysis, the following are the top 5 variables that predicted a need for preoperative embolization: BMI, Shamblin III, age, current drinking, female.

Variable	Importance score
BMI, kg/m2	42.6
Shamblin III	29.9
Age, years	8.8
Current drinking	8.4
Female	5.1

BMI, Body mass index.

## Discussion

This study indicated that preoperative embolization of CBTs did not reduce perioperative morbidity and mortality. No patient died during follow-up after CBT surgical resection in our current study. There were two patients with stroke in the surgical resection alone group and one in the preoperative embolization group. It seemed to be more risky for patients who received preoperative embolization, although the few occurrences made it difficult to perform meaningful statistical analysis. Notably, this one patient in the preoperative embolization group developed a stroke after the CBT surgical resection. There was no direct evidence to prove that preoperative embolization was associated with stroke in the current study.

Furthermore, the LOO in group B (152.9 ± 40.4 min) seemed to be longer than that in group A (132.6 ± 64.6, min) and there was no significant difference for IBL between the two groups. We speculated that preoperative embolization would make the tumor more tenacious, which increased the surgical difficulty and resulted in a longer LOO and more IBL. Notably, the LOS in group B (7.4 ± 1.7 day) was less than that in group A (8.3 ± 2.0 day). However, LOS in this current study did not include the time when patients were first hospitalized for preoperative embolization.

In most cases, CBTs are slowly developing and remain asymptomatic for several years (17). Several researches reported that significant blood loss is associated with severe complications for CBT patients who received surgical resection (18–20). The purpose of preoperative embolization is to reduce surgical blood loss (1). However, it is still controversial whether embolization should be performed before surgical resection of CBT. A meta-analysis showed that the operative parameters and adverse events after surgical resection were not significantly different in CBT patients with or without preoperative embolization (5). Another multi-institutional retrospective study demonstrated no significant difference in the rate of postoperative complications between these two groups, and the operation time was not reduced in Shamblin I and II CBTs (7).

It is also argued that patients who undergo preoperative embolization are more likely to experience stroke than those who undergo surgical resection alone and the cost even outweighs the benefits of decreased blood loss (11, 13). According to previous studies, the rates of stroke in preoperative embolization patients presented 0% and 8% (21, 22). In our current study, the rate of stroke in total patients was 1.1% (0.9% in group A vs. 2.9% in group B) (p = .308). An early study evaluated the efficacy of preoperative embolization, but their results failed to demonstrate any benefit (11). Otherwise, a preoperative embolization may cause an internal carotid embolism, increasing the risk of stroke (18, 23). A recent study also found that preoperative embolization increased intraoperative bleeding in CBT surgical resections (24).

This population has a wide range of CN injuries ranging from 0% to 49% (21). The rate of CN injuries in both groups in our study was 27.6% (28.8% in group A vs. 20.0% in group B) (p = .281). Surgical resection procedure for CBT was commonly performed 1–2 days after preoperative embolization in these centers. No obvious CN injuries were observed following preoperative embolization and before the CBT surgical resection procedure. The results indicated that no direct association was found between CN injuries and preoperative embolization. CN injuries were commonly caused by excessive retraction according to these results and our experience.

Excessive retraction causes hypoglossal and marginal mandibular nerve palsies in most CBT cases (13). In this study, the top three injured CNs after CBT surgical resection were the pharyngeal branch and superior laryngeal nerve, recurrent laryngeal nerve, and vagus nerve. During a 3-year follow-up, most CN injuries were temporary and could be fully recovered.

In our institution, surgical resection of CBT is most commonly performed without preoperative embolization. This study demonstrated that there was insufficient evidence to support the efficacy of preoperative embolization, CBT resection alone had a low rate of death or stoke, recurrence, and CN injuries, and CBT resection alone did not increase LOO and IBL.

## Study limitations

The clinical severity of these CBT cases is evaluated by using the Shamblin classification. Only a part of these larger tumors, like Shamblin II and III, are embolized preoperatively; it is difficult to reliably identify the small subset of patients who would benefit from preoperative embolization of their CBTs. All these CN injuries were verified by patients’ clinical symptoms. This might be the reason for the inconsistency between previous studies and our results. A clinical trial study for CBT might be more convincing. However, the sample size is greatly limited due to the low incidence rate of CBT. Finally, these data are collected from three representative hospitals; this may result in the results not being generalizable to all populations.

## Conclusions

This study demonstrated that there was insufficient evidence to support the efficacy of preoperative embolization. CBT resection alone had a low rate of death or stoke, recurrence, and CN injuries, and CBT resection alone did not increase LOO and IBL.

Surgical management of CBT patients without preoperative embolization is also safe and effective. Preoperative embolization remains a controversial topic.

## Data availability statement

The original contributions presented in the study are included in the article/supplementary material. Further inquiries can be directed to the corresponding authors.

## Author contributions

Conception and design: TH, JP, HT, BZ, DG, XT, ZZ. Analysis and interpretation: TH, JP, HT, SY, DD, ML. Data collection: TH, JP, HT, XW. Writing the article: TH, JP, HT, BZ, DG. Critical revision of the article: TH, JP, HT, SY, DD, ML, XW, GY, BZ, DG, XT, ZZ. Final approval of the article: TH, JP, HT, SY, DD, ML, XW, GY, BZ, DG, XT, ZZ. Agreement to be accountable: TH, JP, HT, SY, DD, ML, XW, GY, BZ, DG, XT, ZZ. Statistical analysis: TH, JP, HT, XW. Obtained funding: GY, BZ. All authors contributed to the article and approved the submitted version.
